# SATB1-dependent mitochondrial ROS production controls TCR signaling in CD4 T cells

**DOI:** 10.26508/lsa.202101093

**Published:** 2021-09-28

**Authors:** Taku Kuwabara, Fumio Ishikawa, Masataka Ikeda, Tomomi Ide, Terumi Kohwi-Shigematsu, Yuriko Tanaka, Motonari Kondo

**Affiliations:** 1 Department of Molecular Immunology, Toho University School of Medicine, Tokyo, Japan; 2 Faculty of Health Sciences, Tsukuba International University, Tsuchiura, Japan; 3 Department of Cardiovascular Medicine, Graduate School of Medical Sciences, Kyushu University, Fukuoka, Japan; 4 Department of Orofacial Science, University of California San Francisco School of Dentistry, San Francisco, CA, USA

## Abstract

Special AT-rich sequence binding protein-1 (SATB1) is localized to the nucleus and remodels chromatin structure in T cells. SATB1-deficient CD4 T cells cannot respond to TCR stimulation; however, the cause of this unresponsiveness is to be clarified. Here, we demonstrate that SATB1 is indispensable to proper mitochondrial functioning and necessary for the activation of signal cascades via the TCR in CD4 T cells. Naïve SATB1-deficient CD4 T cells contain fewer mitochondria than WT T cells, as the former do not express mitochondrial transcription factor A (TFAM). Impaired mitochondrial function in SATB1-deficient T cells subverts mitochondrial ROS production and SHP-1 inactivation by constitutive oxidization. Ectopic TFAM expression increases mitochondrial mass and mitochondrial ROS production and rescues defects in the antigen-specific response in the SATB1-deficient T cells. Thus, SATB1 is vital for maintaining mitochondrial mass and function by regulating TFAM expression, which is necessary for TCR signaling.

## Abstract (precis)

SATB1 regulates mitochondrial function and reactive oxygen species (ROS) production through the expression of mitochondrial transcription factor A. SATB1-mediated ROS production is necessary for TCR stimulation and T-cell function.

## Introduction

T-cell activation is triggered by signals via the TCR upon recognition of the complex composed with antigen peptides and self-MHC (Nel, 2002; Nel & Slaughter, 2002; Smith-Garvin et al, 2009). Lymphocyte-specific protein tyrosine kinase (Lck), an Src family tyrosine kinase, initiates downstream TCR signaling by phosphorylating the immunoreceptor tyrosine-based activation motif (ITAM) within the TCR-associated CD3ζ-chains (Molina et al, 1992; Straus & Weiss, 1992). Phosphorylated ITAM generate docking sites for 70-kD ζ-chain–associated protein kinase (ZAP70). Lck also phosphorylates ZAP70, which propagates signaling events such as intracellular calcium influx and the MAPK kinase known as Ras-MAPK or extracellular signal-regulated kinase (ERK) (van Leeuwen & Samelson, 1999). Both of these events are necessary for T-cell activation (Smith-Garvin et al, 2009; Courtney et al, 2018; Gaud et al, 2018). Thus, regulation of Lck activity is critical for T-cell function.

A major negative regulator of Lck, which sets the TCR signaling threshold, is the tyrosine phosphatase SHP-1 (Kosugi et al, 2001). Aberrant Lck activation is observed in SHP-1–deficient mice leading to T-cell hyperactivation, increased IL-2 production, and autoimmunity (Carter et al, 1999; Lorenz, 2009). Furthermore, the TCR signal cascade cannot be activated in T cells in the presence of the constitutive active form of SHP-1 (Štefanová et al, 2003; Capasso et al, 2010). Therefore, regulation of SHP-1 activity is crucial for T-cell activation. However, the regulatory mechanisms of SHP-1 activity in resting T cells are not well-understood.

Mitochondria are the powerhouses of cells as they produce cellular energy sources such as adenosine 5′-triphosphate (ATP) (Mills et al, 2017). Mitochondria play key roles in the tricarboxylic acid (TCA) cycle and cellular respiration and participate in fatty acid synthesis, Ca^2+^ homeostasis, and heme and Fe-S protein biogenesis (Tait & Green, 2012). For mitochondrial biogenesis, *Tfam*, a nuclear gene encoding mitochondrial transcription factor A (TFAM), is essential for mitochondrial DNA transcription and replication (Fisher et al, 1989; Larsson et al, 1998; Nisoli et al, 2005). In various cell types, the multifunctional mitochondria are integrated into the cell signaling circuitry (Gunter et al, 2004; Chandel, 2010; Finkel, 2011; Arciuch et al, 2012; Tait & Green, 2012). In T cells, mitochondria move near the TCR complex, which appear to be required for T-cell activation (Quintana et al, 2007; Baixauli et al, 2011). However, the precise functional roles of mitochondria in TCR signals remain unclear.

The chromatin structure modulates the accessibility of various transcription factors to the regulatory regions of target genes and regulates gene expression. Such chromatin topology is regulated by chromosomal organizers (Schneider & Grosschedl, 2007; Phillips & Corces, 2009). Matrix attachment DNA regions (MARs) or scaffold-associated regions in DNA sequences may mediate chromatin loop formation, which is essential for genomic DNA compaction and chromatin organization into genomic functional units (Cockerill et al, 1987; Zlatanova & van Holde, 1992). MARs often colocalize with enhancer regions and regulate both positive and negative gene expression (Banan et al, 1997; Zhang et al, 1998; Zhong et al, 1999). Special AT-rich sequence binding protein-1 (SATB1) is an MAR-binding protein (Dickinson et al, 1992) and genome organizer. SATB1 anchors AT-rich DNA sequences to the nuclear matrix and forms genomic DNA loops (Cai et al, 2003; Kumar et al, 2007). SATB1 can also recruit chromatin-remodeling complexes and regulate gene expression (Kumar et al, 2007). SATB1 is necessary for the expression of several T helper genes and modulates effector T-cell function by holding the promoter regions of cytokine genes in close proximity (Cai et al, 2006). In addition, mice without SATB1 in their T cells failed to respond to antigens, albeit SATB1-deficient mice are prone to developing autoimmune disorders (Tanaka et al, 2017; Akiba et al, 2018). Accordingly, SATB1 is essential for T-cell activation; however, its contribution to T-cell function remains unknown.

In this study, we demonstrate that SATB1 regulates mitochondrial reactive oxygen species (mtROS) production and mitochondrial maintenance through the expression of TFAM protein. We also found that SATB1-dependent mtROS is necessary for modulation of SHP-1 activity and threshold setting for T-cell activation upon TCR stimulation. Therefore, SATB1 plays a vital role in TCR function by maintaining mitochondrial homeostasis. The insights obtained from this study will help in the development of treatment strategies for T-cell–mediated immune diseases.

## Results

### SATB1 is indispensable in T-cell activation

**Figure S1.**
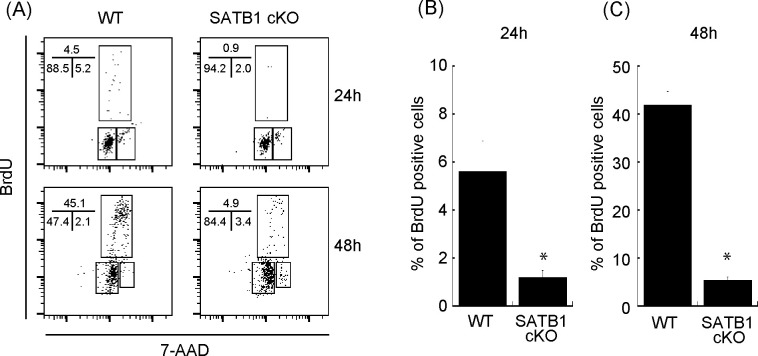
Cell cycle analysis of CD4 T cells from WT and SATB1cKO mice. Sorted naïve CD4 T cells were stimulated with anti-CD3 and anti-CD28 antibodies for 24 and 48 h. BrdU was added to the in vitro culture for the last 30 min of incubation. **(A)** The cells were then fixed and stained with anti-BrdU antibody and 7-amino-actinomycin and subjected to FACS analysis (A). The percentage of cells in each cell cycle phase is shown. Data are representative for three independent experiments. **(B, C)** The quantification of BrdU-positive cells is shown on the right-hand side (B, C).

To determine the relevance of SATB1 protein in T-cell activation, we purified naïve CD4 T cells from wild type (WT) and SATB1cKO mice, stimulated the cells with anti-CD3 and anti-CD28 antibodies, and examined the activation status via the bromodeoxyuridine (BrdU) uptake and DNA content. We found that 4.5% and 45% of T cells from WT mice were BrdU-positive at 24 and 48 h after TCR stimulation, respectively. In contrast, T cells from SATB1cKO mice contained far fewer BrdU-positive cells (Fig S1A–C). Hence, SATB1 is required for T-cell proliferation by TCR-mediated stimulation in accordance with our previous reports (Tanaka et al, 2017; Akiba et al, 2018).

**Figure 1.**
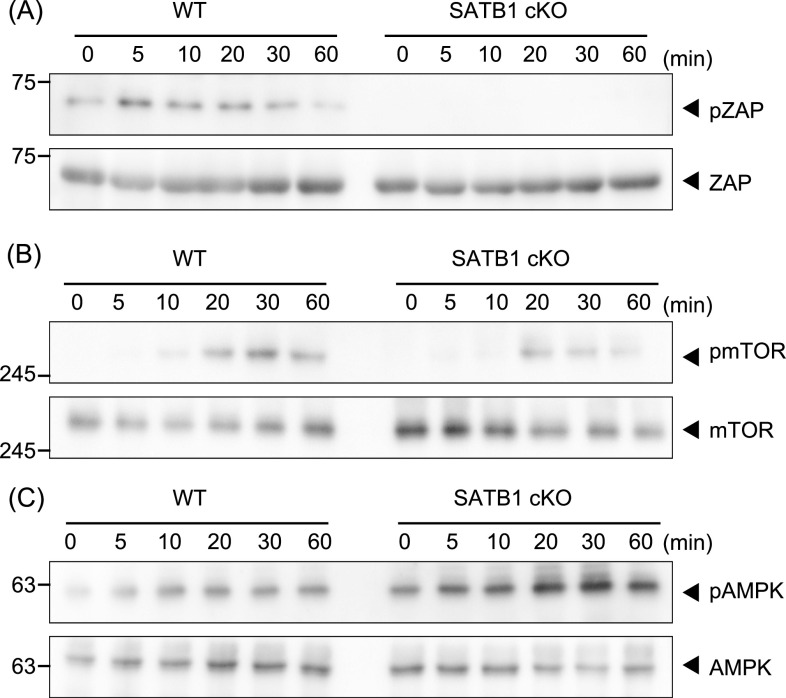
SATB1 is required for T-cell activation. Signaling molecule phosphorylation was examined in naïve CD4 T cells from WT and SATB1cKO mice. **(A, B, C)** T cells were stimulated with anti-CD3 and anti-CD28 antibodies for the indicated time periods, lysed, analyzed by SDS–PAGE, and immunoblotted with anti-ZAP70, anti-phospho-ZAP70 (A), anti-mammalian target of rapamycin, anti-phospho-mammalian target of rapamycin (B), anti-AMPK, and anti-phospho-AMPK (C). Data for one experiment are representative of three independent experiments.

Next, we investigated whether SATB1-deficient T cells adequately phosphorylate the signaling molecules in the TCR cascade. In WT T cells, we detected ZAP70 phosphorylation in TCR-mediated signaling within 5 min of cross-linking with anti-CD3 and anti-CD28 antibodies (Fig 1A). However, T cells from SATB1cKO mice showed substantially lower ZAP70 phosphorylation than the WT control. These results demonstrate that SATB1 is necessary for triggering TCR signal cascades in CD4 T cells.

### Mitochondrial function is impaired in SATB1cKO T cells

**Figure S2.**
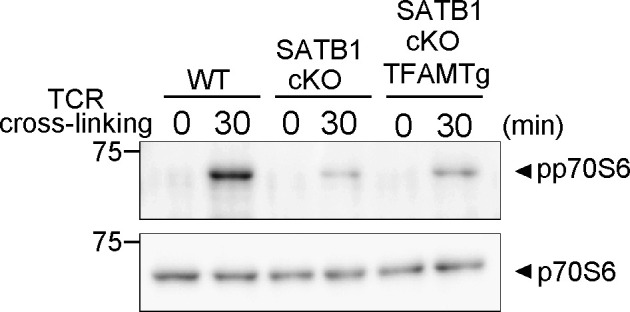
Signaling molecule phosphorylation examined in naïve CD4 T cells from WT, SATB1cKO, and SATB1cKO-TFAMTg mice. T cells were stimulated with anti-CD3 and anti-CD28 antibodies for the indicated periods, lysed, analyzed by SDS–PAGE, and immunoblotted with antibodies against p70S6 and phospho-p70S6 kinases. Data for one experiment representative of three independent experiments are shown.

The mammalian target of rapamycin (mTOR) pathway plays an important role in T-cell activation (MacIver et al, 2013; Desdin-Mico et al, 2018). In accordance with the results shown above, mTOR phosphorylation was markedly reduced in T cells from SATB1cKO mice than those from WT mice after TCR stimulation (Fig 1B). In addition, phosphorylation of p70S6 kinase, a downstream molecule of mTOR was much lower in SATB1cKO CD4 T cells than in WT CD4 T cells (Fig S2). Because tonic signals via TCR are necessary for T-cell survival, we hypothesized that SATB1cKO CD4 T cells were under energetic stress, leading to activation of the serine/threonine AMP-activated protein kinase (AMPK). AMPK can be phosphorylated in T cells upon TCR stimulation. In fact, we observed increased AMPK phosphorylation levels in both WT and SATB1cKO T cells (Fig 1C). More importantly, AMPK was phosphorylated in the resting state of SATB1cKO cells but not WT T cells (Fig 1C), suggesting that SATB1cKO T cells lack sufficient cellular energy, in which AMPK may be constitutively activated.

**Figure 2.**
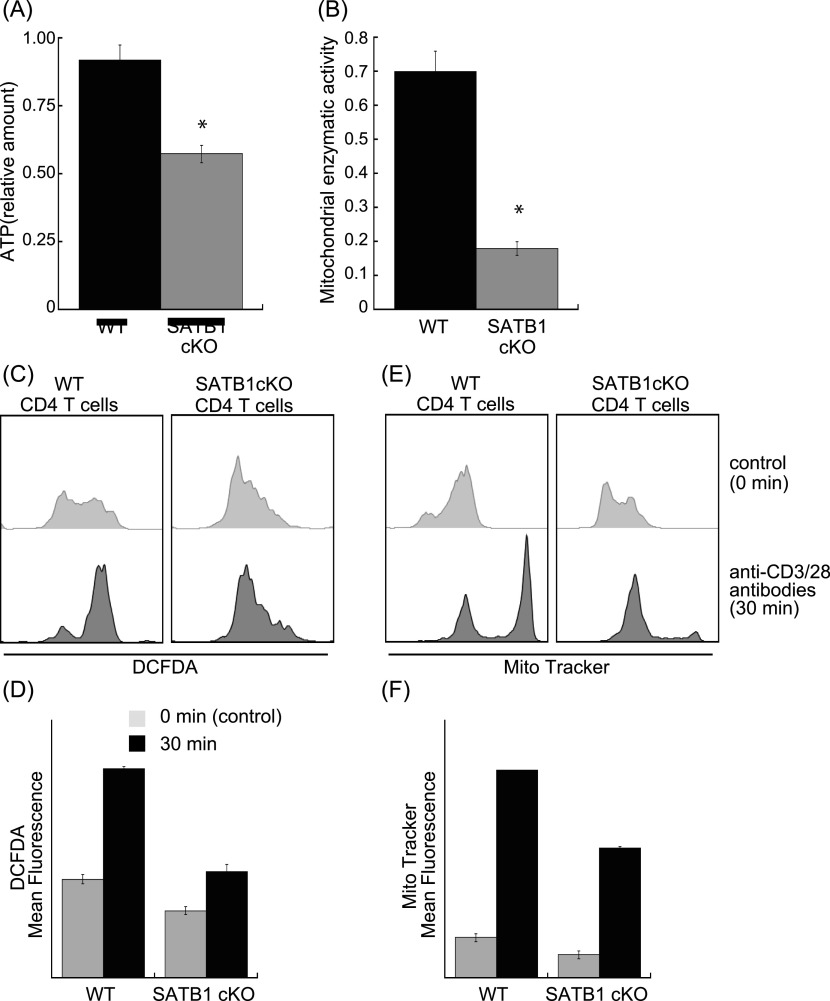
SATB1-TFAM axis is required for mitochondrial homeostasis. **(A)** CD4 T cells were suspended in ATP assay buffer and incubated for 1 min at RT. Light intensity was indicative of intracellular ATP content (Lu-ATP). **(B)** Oxidative phosphorylation complex I enzyme activity was measured. Lysates were incubated on the plate for 3 h to capture complex I. Enzyme activity was determined by NADH oxidation to NAD+ and simultaneous dye reduction. Dye absorbance was measured with a plate reader at 450 nm. **(C)** Levels of mtROS in CD4 T cells from WT, and SATB1cKO mice in the control and after TCR activation were measured by DCFDA staining and flow cytometry. **(D)** Quantification of mean fluorescence of DCFDA. **(E)** Representative MitoTracker staining histograms. WT and SATB1cKO T cells in the control and after TCR activation were stained with MitoTracker and analyzed by a flow cytometer. **(F)** Quantification of the mean fluorescence of MitoTracker.*P < 0.01 versus WT. N = 5. Data are shown as the means ± SD.

**Figure S3.**
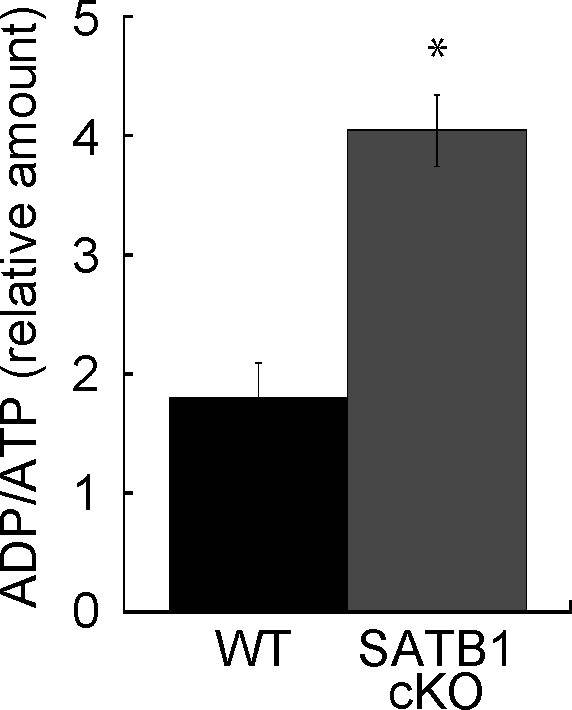
ADP/ATP ratio in T cells. Cellular ATP was assessed. Cell lysates were incubated with ADP assay buffer for 1 min and luminescence was measured (Lu-ADP). ADP/ATP ratio was calculated as Lu-ADP/Lu-ATP. Dye absorbance was measured in a plate reader at 450 nm. *P < 0.01 versus WT. N = 5. Data are shown as the means ± SD.

Cellular processes such as transcription and translation are enhanced in T cells after TCR stimulation. T-cell activation requires sufficient cellular energy such as ATP, suggesting that loss of mitochondria and short supply of ATP occur in SATB1-deficient T cells. Next, we examined the cellular ATP levels in CD4 T cells from WT and SATB1cKO mice. Primary CD4 T cells from SATB1cKO mice contained 60% less ATP than those from WT mice. The former also showed increased ADP/ATP ratios (Figs 2A and S3). Thus, SATB1 loss may cause severe energetic stress and mitochondrial dysfunction. Therefore, we investigated whether mitochondrial function is weaker in SATB1cKO than in WT T cells. The activity level of an NADH dehydrogenase in mitochondrial complex I was markedly lower in SATB1-deficient T cells than in WT T cells (Fig 2B). We prepared naïve CD4 T cells from WT and SATB1cKO mice, incubated them with the ROS-sensitive dye DCFDA, and measured intracellular ROS levels. As shown in Fig 2C, WT T cells had a distinct bimodal fluorescence distribution in the absence of TCR stimulation. In contrast, SATB1cKO CD4 T cells exhibited low unimodal fluorescence peaks (Fig 2C). TCR stimulation for 30 min increased the relative DCFDA fluorescence-positive WT T-cell population (Fig 2C and D). However, no change in the DCFDA-positive population was observed in SATB1cKO T cells after TCR stimulation (Fig 2C and D), demonstrating that TCR activation promotes mtROS production in WT but not in SATB1-deficient T cells. CD4 T cells were also incubated with MitoTracker. Similar to DCFDA staining, WT CD4 T cells after TCR activation increased the proportion of MitoTracker-positive cells. In contrast, the MitoTracker-positive population was less in SATB1cKO T cells after TCR stimulation (Fig 2E and F). Therefore, SATB1 is vital for proper mitochondrial function and necessary for mtROS production in T cells even in the resting state.

### SATB1-deficient T cells show high SHP-1 activity

**Figure 3.**
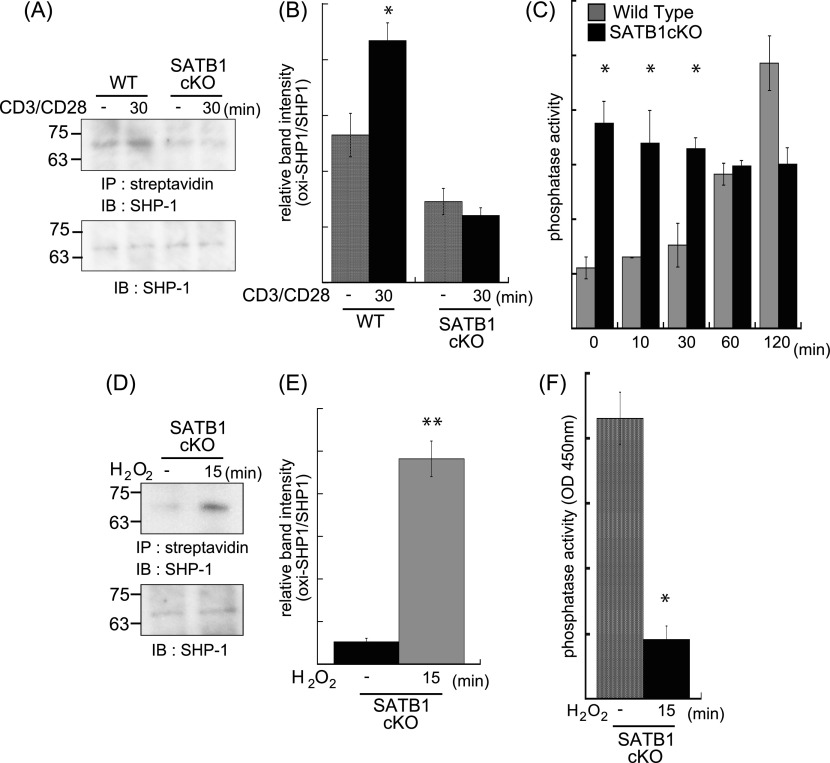
Phosphatase SHP-1 is regulated by mtROS. WT and SATB1cKO naïve CD4 T cells were stimulated with anti-CD3 and anti-CD28 antibodies for the indicated times and lysed in sample buffer. Oxidized proteins in the cell lysates were labeled with iodoaceto-PEG biotin and immunoprecipitated. **(A)** Blots were probed for anti–SHP-1 antibody. **(B)** Band intensities were quantified with ImageJ v. 1.49. **(C)** Naïve CD4 T Cells were incubated for 0, 10, 20, 30, 60, and 120 min with anti-CD3 and anti-CD28 antibodies and lysed in phosphatase assay buffer. Phosphatase activity was assayed by incubation with PY containing peptide and liberated phosphate was measured by malachite green absorption at 450 nm. **(D, E)** Lysates of SATB1-deficient naïve CD4 T cells were incubated in the absence or presence of H_2_O_2_ for 15 min. Oxidized SHP-1 was detected by immunoblotting with antibody against SHP-1 (D). **(E)** Band intensities were quantified with ImageJ v. 1.49 (E). **(B, F)** Phosphatase activity in the presence or absence of H_2_O_2_ was measured in (B). Data are shown as the means of three experiments performed in duplicate. *P < 0.01 versus WT. **P < 0.05 versus WT. N = 5. Data are shown as the means ± SD.

mtROS inactivates receptor-mediated signaling molecules such as phosphatases by oxidization, thereby enhancing and stabilizing kinase cascades (Meng et al, 2002; Kwon et al, 2004; Persson et al, 2004; Crump et al, 2012). As mitochondria localize near the TCR, mtROS may influence the TCR cascade. To determine whether mtROS oxidize phosphatases in TCR cascades, we investigated the oxidization status of SHP-1. Oxidized SHP-1 was weakly detected under basal conditions (0 min) and clearly observed after TCR cross-linking (30 min) in naïve CD4 T cells from WT mice (Fig 3A and B). In contrast, SATB1cKO T cells showed reduced oxidative SHP-1 modification under both resting and stimulated conditions (Fig 3A and B). Next, to clarify the relationship between oxidation and phosphatase activity in SHP-1, we examined SHP-1 phosphatase activity in WT and SATB1cKO T cells before and after TCR stimulation. WT T cells showed low activity in the absence of TCR stimulation and gradual increases in the phosphatase activity at 60 and 120 min after TCR cross-linking (Fig 3C). In contrast, SATB1cKO T cells exhibited consistently high SHP-1 activity in both the absence and presence of TCR cross-linking (Fig 3C). These results suggest that oxidation inhibits SHP-1 phosphatase activity. To further explore this issue, we treated T-cell lysates with H_2_O_2_ and investigated whether mtROS-mediated oxidation suppresses SHP-1 function. SHP-1 in the cell lysates was oxidized by treatment with H_2_O_2_ (Fig 3D and E). As predicted, phosphatase activity in SHP-1 derived from SATB1cKO T cells was significantly decreased by oxidation with H_2_O_2_ treatment (Fig 3F). These results indicate that SHP-1 in T cells is constitutively activated in the absence of SATB1 because of the lack of oxidation due to inability of mtROS generation. The results also suggest that SHP-1 oxidation is necessary for suppressing SHP-1 activity, which requires proper triggering of signaling cascades upon TCR stimulation.

### SATB1-deficient T cells have defective mitochondrial maintenance

**Figure 4.**
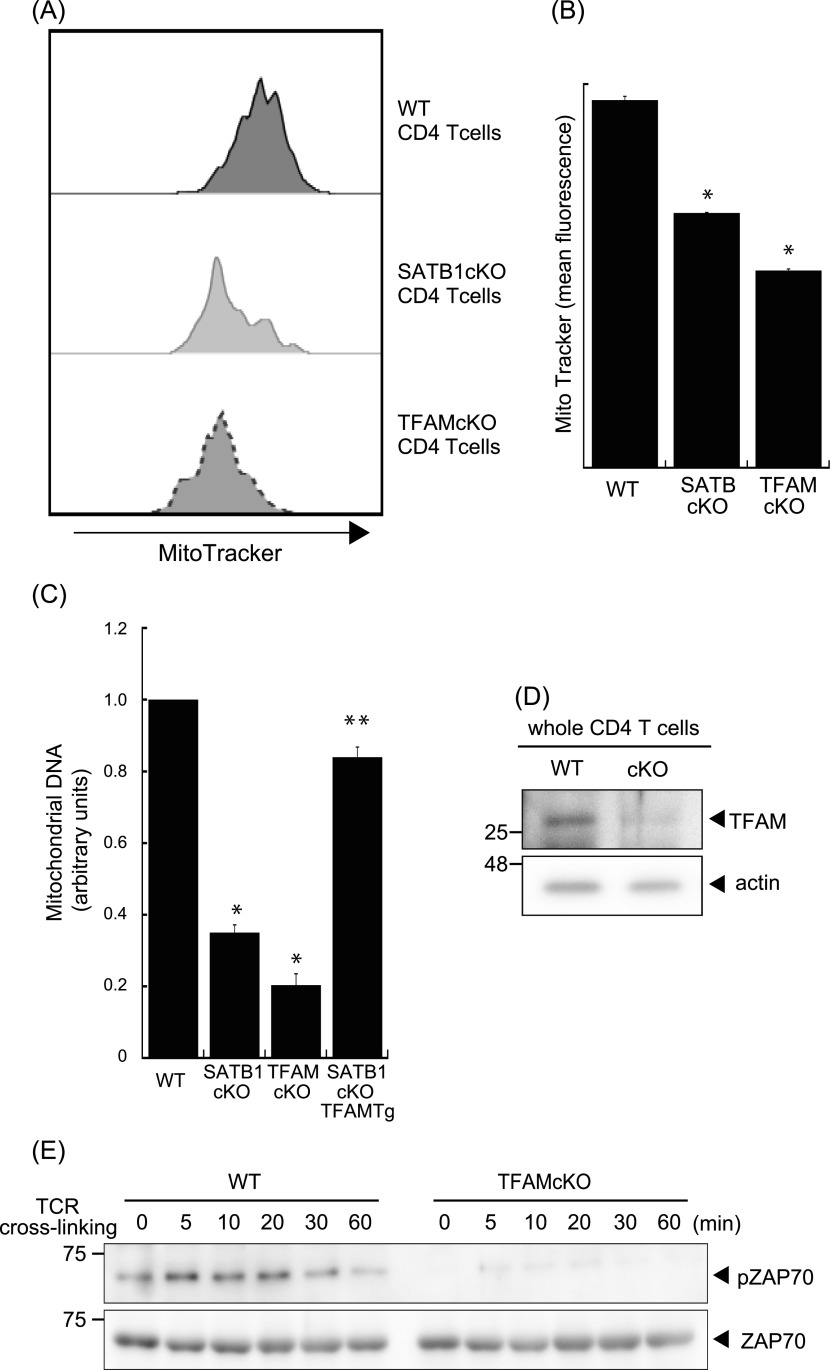
Loss of TFAM expression reduces T-cell activity. **(A)** Representative MitoTracker staining histograms. WT and SATB1cKO T cells were stained with MitoTracker and analyzed by FACS. **(B)** Quantification of the mean fluorescence of MitoTracker is shown. **(C)** Mitochondrial DNA copy numbers in CD4 T cells from WT, SATB1 cKO, TFAMcKO, and SATB1cKO-TFAMTg mice were measured by RT-qPCR. The mitochondrial DNA level was normalized to the nuclear DNA level in each sample. The normalized index is indicated as an arbitrary unit (WT = 1). **(D)** TFAM and β-actin levels in CD4 T-cell lysates were analyzed by immunoblotting using the respective antibodies. **(E)** CD4 T cells were prepared from WT and TFAMcKO mice, stimulated for the indicated times with anti-CD3 and anti-CD28 antibodies, lysed, analyzed by SDS–PAGE, and immunoblotted with antibodies against phospho-ZAP70, and ZAP70. Similar results were obtained for three independent experiments. *P < 0.01 versus WT. **P < 0.01 versus SATB1cKO T cells. N = 5. Data are shown as the means ± SD.

**Figure S4.**
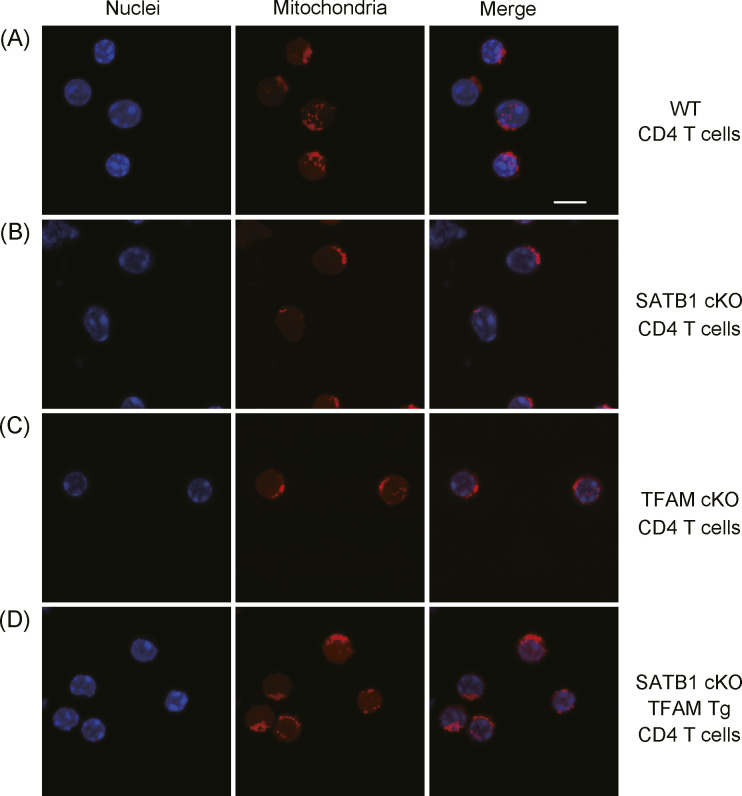
Confocal imaging of mitochondria. **(A, B, C, D)** WT CD4 T cells (A), SATB1cKO CD4 T cells (B), TFAMcKO CD4 T cells (C), and SATB1cKO-TFAMTg CD4 T cells (D) were stained with MitoTracker (mitochondria) and TO-PRO3 dye (nuclei) and analyzed by confocal microscopy. Scale bar, 10 μm. Data represent three independent experiments.

SATB1cKO T cells showed reduced ATP and mtROS production (Fig 2). Thus, we hypothesized that mitochondria in SATB1cKO T cells are dysfunctional. To address this issue, we explored whether SATB1 deficiency in T cells decreases the mitochondrial content. We enumerated the mitochondria by staining them with MitoTracker. FACS-based staining intensity measurement and confocal imaging revealed fewer mitochondria in SATB1cKO T cells than in WT T cells (Figs 4A and B and S4A–D). Moreover, a drastic reduction in mitochondrial DNA confirmed that SATB1cKO T cells had relatively fewer mitochondria (Fig 4C).

**Figure S5.**
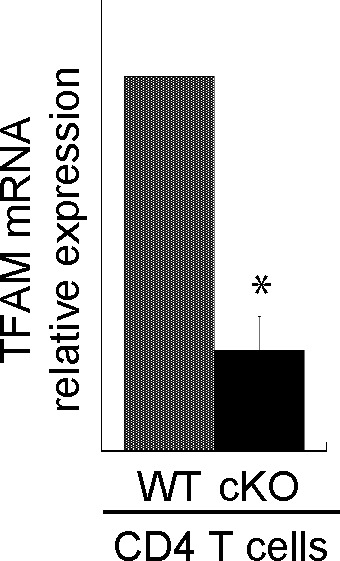
TFAM mRNA expression was analyzed by RT-qPCR. TFAM mRNA transcription levels were analyzed relative to β-actin. Data are shown as the means ± SD for three experiments. *P < 0.01 versus WT.

TFAM plays a role in the maintenance of mitochondrial DNA and homeostasis (Ekstrand et al, 2004; Xu et al, 2009). Thus, we hypothesized that a relationship might exist between SATB1 and TFAM. We evaluated the level of TFAM using an anti-TFAM antibody. Compared with WT CD4 T cells, SATB1-deficient T cells expressed lower levels of TFAM protein (Fig 4D). Similar results were observed at the transcription level (Fig S5). These results suggest that SATB1 regulates TFAM expression.

T cells with disrupted TFAM expression presented with defective mitochondria (Fig 4A) and mitochondrial DNA (Figs 4C and S4). Therefore, down-regulated TFAM may cause defects in mitochondrial function and a loss of T-cell activity in SATB1cKO T cells. To test this hypothesis, we stimulated CD4 T cells from WT and TFAMcKO mice with anti-CD3 and anti-CD28 antibodies and analyzed the cell lysates by immunoblotting. ZAP70 phosphorylation was comparatively reduced in TFAMcKO T cells (Fig 4E). These results indicate that the quiescent phenotype of TFAMcKO T cells resembles that of SATB1-deficient T cells and that TFAM contributes to T-cell activation.

### Ectopic TFAM expression rescues defective TCR signaling in SATB1-deficient T cells

**Figure 5.**
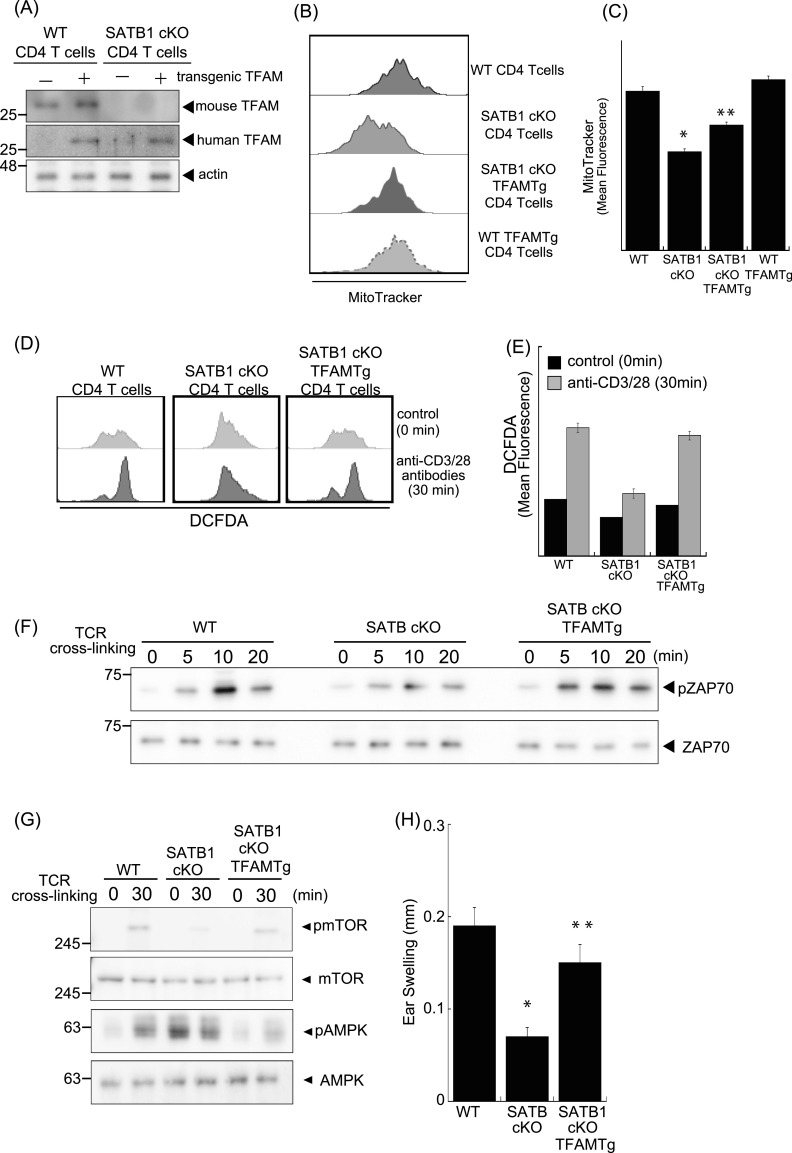
TFAM expression rescues T-cell activity in SATB1cKO mice. **(A)** Immunoblot analysis of transgenic TFAM and endogenous TFAM in SATB1-sufficient or SATB1-deficinet CD4 T cells. **(B)** Representative MitoTracker staining histograms. WT, SATB1cKO, SATB1cKO-TFAMTg, and WT-TFAMTg T cells were stained with MitoTracker and analyzed by FACS. **(C)** Quantification of the mean fluorescence of MitoTracker. *P < 0.01 versus WT. **P < 0.01 versus SATB1cKO T cells. N = 5. Data are shown as the means ± SD. **(D)** ROS levels in CD4 T cells from WT, SATB1cKO, and SATB1cKO-TFAMTg mice in control and TCR activation treatments were measured by DCFDA staining and flow cytometry. **(E)** Quantification of the mean fluorescence of DCFDA. **(F, G)** CD4 T cells were prepared from WT, SATB1cKO, and SATB1cKO-TFAMTg mice and functionally analyzed. T cells were stimulated with anti-CD3 and anti-CD28 antibodies for the indicated times, lysed, analyzed by SDS–PAGE, and immunoblotted with antibodies against phospho-ZAP70, ZAP70, phospho-mammalian target of rapamycin, phospho-AMPK, and AMPK. Similar results were obtained for three independent experiments. **(H)** Mice were sensitized with 7% PCI in acetone/olive oil. After 7 d, 1% (vol/vol) PCI was applied to the right ear and vehicle alone was applied to the left ear. Ear thickness was measured after 24 h. Means ± SD for 4–5 mice are indicated. *P < 0.01 versus WT. **P < 0.01 versus SATB1cKO T cells. N = 5. Data are shown as the means ± SD.

To further address whether TFAM expression complements SATB1 deficiency in T cells, we generated SATB1cKO mice with an ectopic TFAM gene. Transgenic human TFAM expression under SATB1-sufficient or SATB1-deficient conditions was detected as similar protein levels (Fig 5A). The mitochondrial mass and DNA levels in T cells from SATB1cKO-TFAM transgenic (Tg) mice were restored nearly to those in WT T cells (Figs 4C, 5B and C, and S4). Decreased mtROS production in the steady state was also restored, and DCFDA fluorescence-positive population induced by TCR stimulation was rescued in T cells from SATB1cKO-TFAMTg mice (Fig 5D and E).

Active ZAP70 induced by TCR cross-linking was reconstituted to nearly normal levels in CD4 T cells from SATB1cKO-TFAMTg mice (Fig 5F). In addition, the mTOR and p70S6 kinase phosphorylation patterns induced by TCR stimulation were recovered (Figs 5G and S2). The AMPK phosphorylation observed in T cells lacking SATB1 expression was absent in T cells from SATB1cKO-TFAMTg mice in the resting state and induced upon TCR stimulation (Fig 5G). However, the level of phosphorylation in SATB1cKO-TFAMTg T cells was lower than WT T cells, suggesting that other SATB1-target genes than TFAM might be involved in proper AMPK phosphorylation pathway. Nevertheless, these results suggest that defective T-cell response in the absence of SATB1 might be due to lack of TFAM expression.

Finally, we examined whether ectopic TFAM expression could recover impaired T-cell function in SATB1cKO mice in vivo by the picryl chloride (PCI) contact dermatitis model. This inflammatory skin disease is mediated by hapten specific T cells (Asherson & Ptak, 1968; Austrup et al, 1997; Tietz et al, 1998). SATB1cKO mice showed less ear swelling compared with WT mice after re-challenged with PCI (Fig 5H). In contrast, the contact hypersensitive response was rescued in SATB1cKO mice with exogenous TFAM expression. This result indicates that the T-cell response was intact in SATB1cKO-TFAMTg mice. Taken together, our results demonstrate that SATB1 plays a significant role in T-cell activation by maintaining mitochondria.

## Discussion

In this study, we demonstrated that SATB1cKO T cells failed to express TFAM, maintain the mitochondria, and respond to TCR stimuli. Enforced TFAM expression rescued the activation capability of SATB1-deficient T cells in response to TCR stimuli, indicating that lack of TFAM expression, which leads to loss of mitochondria contents, is largely responsible for T-cell dysfunction in SATB1cKO mice. Based on these findings, it seems that the role of SATB1 in T-cell activation is to maintain the mitochondria through TFAM expression in T cells.

TCR stimulation induces activation of SHP-1 by phosphorylation at its tyrosine residues (Zhang et al, 2000). As we showed in this study, SATB1cKO T cells possess high SHP-1 activity, even in the resting state. In addition, tyrosine phosphorylation levels of signal molecules proximate to TCR were extremely low. Therefore, SHP-1 activation is enhanced in SATB1-deficient T cells by other mechanisms than phosphorylation. SATB1-deficient T cells showed a smaller number of mitochondria and lower production of ATP than in WT T cells. The metabolic regulator AMPK was also activated in SATB1-deficient T cells in the resting state. These results suggest that AMPK-mediated machinery may activate SHP-1 in SATB1-deficient T cells. AMPK activation also suggested that metabolic dysfunction (low amount of ATP) in SATB1-deficient T cells. As cellular ATP is necessary for T-cell activation (van der Windt et al, 2012; Pearce et al, 2013; Maciolek et al, 2014), metabolic stress interferes to induce T-cell activation in SATB1-deficient T cells. We should note that these results are not sufficient to determine either AMPK-mediated SHP-1 activation or energy stress is the causative reason of T-cell dysfunction in SATB1-deficient T cells.

SATB1 is a molecular adapter for chromatin-remodeling complexes that tighten or loosen chromosomal DNA into an active or inactive state (Yasui et al, 2002). SATB1 forms a unique transcriptional chromatin structure and up-regulates genes of Th2 cytokines (Cai et al, 2006). As SATB1 regulates the activity of genes near its DNA-binding sites, we used a ChIP assay with anti-SATB1 antibodies to detect SATB1 binding to the regulatory regions of *Tfam*. However, there was no evidence that SATB1 binds to the *Tfam* gene (data not shown). *Tfam* expression is regulated by various transcription factors such as PGC-1α and NRF (Piantadosi & Suliman, 2006; Kozhukhar & Alexeyev, 2019). Thus, SATB1 may indirectly up-regulate the transcriptional activity of *Tfam*. SATB1 may also associate with various proteins, such as β-catenin and up-regulate genes in activated T cells (Notani et al, 2010). Thus, identification of the SATB1 binding partner may reveal the regulatory mechanisms of *Tfam* expression.

Mitochondrial activities are known to be linked to T-cell functions (Bantug et al, 2018; Desdin-Mico et al, 2018; Zhu & Thompson, 2019). T cells sense extracellular chemotactic factor gradients that recruit them to infection or inflammatory sites and cause various changes in T-cell morphology (Campello et al, 2006). The cell shape change indicates polarization that initiates the formation of leading edges and uropods. Mitochondria accumulate at uropods and provide ATP during directed T-cell migration (Campello & Scorrano, 2010). After clearing invaded pathogens, most effector T cells diminish during the contraction phase, whereas memory T cells employ their activities such as oxidation lipid metabolism (Bantug et al, 2018; Desdin-Mico et al, 2018; Zhu & Thompson, 2019). Memory T cells display an increased mitochondrial number and maximal mitochondrial respiratory capacity (Pearce et al, 2009; van der Windt et al, 2012). Therefore, SATB1 may be involved in maintaining effector T-cell potential and supporting mitochondrial activity.

Here, we demonstrated that SATB1 modulates TCR signaling. To the best of our knowledge, this is the first study to demonstrate that SATB1-mediated mtROS production is required to generate T-cell responses. mtROS from mitochondria regulated by SATB1 maintain ZAP70 and ERK activation downstream of TCR via oxidative SHP-1 inhibition. Therefore, SATB1 is a key molecule in mitochondrial activity and is essential for T-cell homeostasis.

## Materials and Methods

### Mice

SATB1-floxed mice and transgenic mice expressing mitochondrial transcription factor A (TFAM) were previously described (Ikeuchi et al, 2005; Kondo et al, 2016). Vav-Cre and TFAM-floxed mice were purchased from The Jackson Laboratory. Lck-Cre mice were obtained from the Laboratory Animal Resource Bank at NIBIOHN. SATB1 or TFAM conditional knockout mice were generated by crossing floxed, Vav-Cre, and Lck-Cre mice. All mice were maintained on a C57BL/6 background under specific pathogen-free conditions at the Toho University School of Medicine animal facility (Kuwabara et al, 2009). All mouse experiments were approved by the Toho University Animal Care and User Committee (No. 20-51-435) and Toho University Safety Committee for Recombinant DNA Experiments (No. 20-51-440). Experiments were performed on 8- to 12-wk-old mice.

### Antibodies

Anti-SHP-1 was purchased from Abcam. Anti-AMPK, anti-phosphorylated-AMPK, anti-ZAP70, anti-phosphorylated-ZAP70, anti-mTOR, anti-phosphorylated-mTOR, anti-p70 S6 kinase, and anti-phosphorylated-p70 S6 kinase antibodies were acquired from Cell Signaling Technology.

### Mitochondrial DNA

qPCR quantified the mitochondrial DNA copy number. Total DNA was isolated with a DNeasy blood and tissue kit (QIAGEN GmbH) according to the manufacturer’s instructions. Mitochondrially encoded nicotinamide adenine dinucleotide NADH dehydrogenase 1 (mND1) and hexokinase gene 2 (NK2) DNA were amplified with Maxima SYBR Green qPCR Master Mix (Thermo Fisher Scientific). To quantify the mitochondrial DNA copy number, the ratio of mitochondrial ND1 to nuclear HK2 was calculated by the 2^−ΔΔCt^ method. The primers used were forward 5′-CTAGCAGAAACAAACCGGGC-3′ and reverse 5′-CCGGCTGCGTATTCTACGTT-3′ for ND1 and forward 5′-GCCAGCCTCTCCTGATTTTAGTGT-3′ and reverse 5′-GGGAACACAAAAGACCTCTTCTGG-3′ for HK2.

### Cell imaging

CD4 T cells from the spleen were prepared by cell sorting and incubated in RPMI-1640 medium supplemented with 10% (vol/vol) FCS and 50 μM of 2-ME for 60 min followed by addition of MitoTracker (Invitrogen) for 20 min. The cells were washed with ice-cold PBS and fixed with 4% (vol/vol) PFA in PBS for 10 min at 25°C. The nuclei were stained with 1 μM TO-PRO3 (Invitrogen) for 20 min at 25°C. The cells were observed under a confocal laser-scanning microscope (LSM510; Carl Zeiss AG). Fluorescence images were processed with Adobe Photoshop (Adobe Systems, Inc.).

### Contact hypersensitivity response

Mice were epicutaneously sensitized on their shaved abdomens with 150 μl of 7% (vol/vol) 2,4,5-trititrochlorobenzene (picryl chloride [PCI]; NacalaiTesque, Kyoto, Japan) in 4:1 (vol/vol) acetone/olive oil. 6 d after sensitization, the right ears were challenged with 20 μl of 1% (vol/vol) PCI in the vehicle. The left ears were treated with vehicle alone. After 24 h, ear thickness was measured with a dial thickness gauge (Dial Caliper, Mitutoyo). Ear thickness was calculated according to the following formula: ear thickness = (ear thickness after 24 h elicitation; right ear) − (control ear thickness; left ear).

### Respiratory enzyme activity

Mitochondrial complex I-driven respiratory capacity was measured in murine CD4 T cells from WT and SATB1cKO mice. Specific rotenone-sensitive NADH oxidoreductase activity was measured with a Complex I enzyme activity microplate assay kit (Abcam) according to the manufacturer’s instructions.

### Immunoblotting

Immunoblotting was performed as previously described (Kuwabara et al, 2012; Matsui et al, 2018). T cells were incubated at 37°C for 4 h in FCS-free RPMI-1640 for cell cycle synchronization. The cells were washed and incubated at a density of 2.0 × 10^6^/ml and stimulated with plate-bound anti-CD3 and anti-CD28 antibodies for the times indicated in Figs and Figs 1 3 –Figs 1 5. Cell extracts were generated from cultured cells with extraction buffer (50 mM Tris–HCl [pH 7.4], 1% [vol/vol] Triton X-100, 450 mM NaCl, 1 mM EDTA, and 1 mM DTT) and proteinase inhibitors. Lysates were centrifuged at 12,000*g* for 10 min at 4°C. Protein concentrations in the supernatants were determined by bicinchoninic acid (Thermo Fisher Scientific) protein assay. Samples were suspended in 2× sample buffer (75 mM Tris–HCl [pH 6.8], 10% [vol/vol] glycerol, 2% [vol/vol] SDS, 0.05% [wt/vol] bromophenol blue, and 2.5% [vol/vol] 2-ME). They were then loaded onto 7.5% (wt/vol) or 10% (wt/vol) SDS–PAGE gels and transferred to polyvinylidene difluoride filters in transfer buffer (25 mM Tris, 0.192 M glycine, and 20% [vol/vol] methanol). The filters were then blocked with 1% (vol/vol) BSA in Tris-buffered saline containing 0.05% (vol/vol) Tween 20 at 25 degrees for 2 h, incubated with the indicated antibodies, incubated with anti-mouse IgG or anti-rabbit IgG coupled with HRP, and visualized with an enhance chemiluminescence detection system (GE Healthcare). Quantitation was performed with ImageJ v. 1.49 software (National Institute of Health).

### Flow cytometry

Antibodies used for flow cytometry [CD4(GK1.5), CD8(53-6.7), B220(RA3-6B2), CD44(IM7), and CD62L(MEL-14)] were purchased from BioLegend. Erythrocytes were depleted from a single-cell spleen and lymph node suspension, incubated with a mixture of fluorescence-conjugated antibodies at 4°C for 30 min, and washed three times with FACS buffer (1% [vol/vol] FCS in PBS and 0.05% [wt/vol] NaN_3_). CD4^+^ CD62L^hi^ CD44^lo^ T cells were then enriched with the cell sorter FACSAria III (BD Biosciences).

Mitochondrial mass and cellular ROS were assessed with MitoTracker (Invitrogen) and a DCFDA cellular ROS detection assay kit (Abcam), respectively, according to the manufacturers’ instructions. Flow cytometry analysis of the cell cycle was performed with a BrdU flow kit (BD Biosciences). To assess viability, the cells were stained with 7-amino-actinomycin (BioLegend) according to the manufacturer’s instructions. Data were acquired with the LSR Fortessa X-20 flow cytometer (BD BiosciencesA) and analyzed with Tree Star FlowJo version 10.5.3 (FlowJo LLC).

### Detection of oxidized proteins

Cells were washed with ice-cold PBS and lysed in oxidized lysis buffer (200 mM ammonium bicarbonate [pH 8.0] and 0.1% [vol/vol] SDS). Cell lysates were incubated under anaerobic conditions. Free thiols were masked with 20 mM iodoacetoamide. Oxidized thiols were reduced with 4 mM DTT and labeled with 1 mM iodoaceto-PEG biotin. Biotinylated proteins were detected by immunoblot analysis.

### Phosphotyrosine phosphatase assay

T-cell extracts (50 μg protein) were incubated with 3 μg anti-SHP-1 antibody for 6 h at 4°C in a total volume of 100 μl. Protein-A beads were added to each sample and incubated for 4 h at 4°C with gentle rotation. Samples were centrifuged and washed 6× with 10 mM Tris–HCl buffer (pH 7.4) containing 100 mM DTT. Washed beads were resuspended in 25 μl of 10 mM Tris–HCl buffer (pH 7.4) and incubated with 100 mM tyrosine phosphopeptide NH_2_-RRLIEDAEpYAARG-COOH for 30 min at 25°C using reagents obtained from Upstate Biotechnology. The reaction was terminated with 25°C malachite green solution and incubated for 15 min to allow for color development. Phosphatase activity was measured at 620 nm in a microtiter plate reader (iMark; Bio-Rad Laboratories).

## Supplementary Material

Reviewer comments
